# Physical activity trajectory during pregnancy and associations with maternal fatigue using a growth mixture modeling approach

**DOI:** 10.1038/s41598-024-51648-w

**Published:** 2024-01-10

**Authors:** Shuhan Yan, Hui Jiang, Ziyi Yang, Xinyan Tang, Zihang Chen, Zhifang Chen, Huahua Liu, Feng Zhang

**Affiliations:** 1 School of Nursing, Nantong University, 19 QiXiu Road, Nantong City, Jiangsu Province China; 2 Health School attached to Shanghai University of Medicine & Health Sciences, 200237 Shanghai, China; 3 Affiliated hospital of Nantong university, Nantong, China; 4 Affiliated Maternity and Child Health Care Hospital of Nantong University, Nantong City, Jiangsu Province China

**Keywords:** Fatigue, Health care

## Abstract

The purpose of this study was to investigate the associations of physical activity trajectories with maternal fatigue. Pregnant women provided objectively assessed physical activity data by Pregnancy Physical Activity Questionnaire four times. Fatigue scale-14 was used to assess fatigue during pregnancy. Growth mixture modelling characterized physical activity trajectories across pregnancy. The generalized estimating equations was used to analyze the relationship between different physical activity profiles and fatigue in pregnant women. A total of 626 pregnant women were included in analysis in a teaching hospital in Nantong city. Fatigue (total, mental and physical) was not different between two groups based on total energy expenditure of PA (constantly high vs. constantly low). The pregnant women in “constantly high household PA” group had the higher fatigue compared to “constantly low household PA” (*P* < 0.05) and “constantly medium household PA” (*P* < 0.05). The pregnant women in “constantly high sport PA” group had lower fatigue compared to “constantly low sport PA” (*P* < 0.05). Household PA and sport PA were still an independent influencing factor for fatigue after controlling for confounding variables. Specifically, we observed that higher household PA and lower sport PA were associated with higher fatigue during pregnancy.

## Introduction

Physical activity (PA) is any deliberate muscle movement that uses energy^1^. Certain studies have found that sufficient physical activity during pregnancy significantly reduces the probability of developing anxiety and distress^2^. Meta-analyses, reviews, and recent randomized trials show promising findings regarding the use of PA in treating depression among cancer survivors^3^. In detail, physical activity can be part of work, leisure time, or any other movement in daily life. There are different types of physical activity, but most people tend to focus on one type of activity. Some articles separated physical activity into five types: household/caregiving, sports/exercise, transportation, occupational, and inactivity. Each type of physical activity brings its own health benefits^4^. Pregnant women with employment had higher risk of adverse pregnancy outcomes than those who were not employed^5^. Some studies found a statistically significant decrease in postpartum depressive symptoms among women engaging in aerobic exercise^6^.

Pregnancy fatigue is a commonly reported complaint among childbearing women^7^. Fatigue has physical and mental effects on an individual. Mental fatigue involves exhaustion of cognitive processes, such as attention and memory, while physical fatigue is related to the depletion of physical energy and muscle strength. Pregnancy fatigue could increase rates of pregnant morbidity, cesarean section, preterm birth, and even postpartum depression^8^. However, longitudinal data on maternal fatigue during pregnancy are limited. By exploring various types of physical activity, this study seeks to elucidate the intricate relationship between maternal fatigue and physical activity behaviors. Understanding this relationship has the potential to positively impact maternal mental health.

Physical activities and fatigue have changes at different trimesters of pregnancy. Fewer studies have assessed pregnancy physical activity trajectories over time and subsequent risks of maternal fatigue^9^. To reduce the reliance on conventional methods^10^, growth mixture modeling (GMM) has been suggested for identifying groups with diverse physical activity trajectories^11^. This method has recently been employed in physical activity research with old adults, linking the identified groups to various health outcomes^12^. Both these time periods and discrete time points are different for changes of pregnancy physical activities, which may also be important for maternal fatigue. Therefore, this study also aims to utilize an individual-based trajectory analysis technique to characterize pregnancy PA trajectories, and to examine their associations with maternal fatigue.

## Methods

Our study was a longitudinal study of pregnant women with singleton pregnancy, ongoing since November 2021. Follow-up examinations through mobile phones took place at different stages of pregnancy. Women were enrolled during their first trimester of pregnancy (gestational weeks 11–13). The inclusion criteria at enrollment were: (1) age greater than or equal to 18 years and (2) ability to speak and understand Chinese. Those having (1) cancer, renal, cardiac dysfunction and other types of diseases that may affect physical activity, (2) prenatal diagnosis of depression or fatigue; (3) threatened abortion; or (4) mobility impairment due to injury or illness affecting the limbs were excluded from the study. Threatened abortion participants were removed from the analysis due to their inability to continue physical activity.

Physical activity was assessed using the Pregnancy Physical Activity Questionnaire (PPAQ), a validated self-report questionnaire. The original questionnaire, which has been widely used and validated in many countries^13,14^. Yan Zhang et.al translated the PPAQ into Chinese^15^. The content validity was 0.94; the test–retest reliability was 0.944. Each activity’s intensity is quantified in terms of energy expenditure, which is assessed using metabolic equivalent of task (MET) units (MET-h week^−1^). The total energy expenditure of PA is generated by summing the energy expenditure of 31 activities. In addition, the 31 activities were also categorized into four types: household PA, occupational PA, sport PA, transportation PA.

Maternal fatigue was evaluated by the fatigue scale-14 (FS-14), which was used to assess maternal fatigue severity widely^16,17^. The FS-14 employed in this study comprises 14 items, encompassing both mental and physical fatigue. The score range for physical fatigue is from 0 to 8, while for mental fatigue is from 0 to 6. Referred to as the FS-14, this scale aims to capture the participants’ subjective experience of fatigue over the preceding two weeks through self-evaluation. A higher score on the scale corresponds to a more pronounced level of fatigue. The internal consistency of each item, as measured by Cronbach's alpha, falls within the range of 0.88–0.90, while the split-half reliability is determined to be 0.86^18^. The sensitivity and specificity of the scale are reported as 75.5 and 74.5%, respectively.

We collected information on women and their partners’ age, educational level, household income, parity, history of diabetes, sleep quality and depression score at recruitment. Height and weight were measured using a portable stadiometer and scales. The Pittsburgh Sleep Quality Index was used to measure maternal sleep quality^19^. Maternal depressive symptoms were evaluated utilizing the Edinburgh Postnatal Depression Scale^20^.

Pregnancy physical activity and fatigue underwent repeated measurements at four-time points: T1 (1st trimester 11–13 gestational weeks), T2 (2nd trimester 20–22 gestational weeks), T3 (3rd trimester 30–32 gestational weeks) and T4 (full-term after 37 gestational weeks). We used these data to identify different physical activity profiles throughout pregnancy.

Sample size calculations were performed using PASS 15.0. Based on previous study, a statistical difference was observed in the fatigue score between different exercise groups (3.52 ± 1.1 vs 3.08 ± 1.0, *P* = 0.04)^21^. We calculated the sample size based on the means and standard deviations of fatigue score between two groups. A sample size of 200 for each of the two groups achieves 80% power with a significance level (alpha) of 0.05. Given a dropout of 20%, the sample size was fixed at 500 participants.

GMMs were estimated using M plus 8.0 to identify trajectories of physical activities across pregnancy. The extraction of profiles as part of GMM was conducted using M plus 8.0. The best-fit model is based on model fit indices: (1) the Akaike Information Criterion (AIC), Bayesian Information Criterion (BIC), and adjusted Bayesian information criterion (aBIC) are smaller; (2) the entropy values are closer to 1; (3) Lo–Mendell–Rubin likelihood ratio test (LMR) and bootstrap likelihood ratio test (BLRT) are both significant. Collectively, the 2-class model was identified based on total energy expenditure of PA. Subsequently, the model with the greatest parsimony, encompassing various types of PA trajectories, was identified (see Supplementary Methods).

Analyses were performed using SPSS 25.0. The selected groups of physical activities (in accordance with the profiles distinguished through GMM) were compared in terms of the results of maternal fatigue. Generalized estimating equations (GEE) were also used to examine the inter-relationship between pregnancy physical activity and maternal fatigue over time. Based on clinical experience and previous reports, covariates potentially associated with PA and fatigue were controlled for. Confounders were determined by a directed acyclic graph (DAG). Finally, maternal age^22,23^, household income^24^, education level, occupation, sedentary employment hours, moderate to vigorous employment hours, parity^25^, depression, sleep quality^26^, IVF, pre-pregnancy BMI, and gestational diabetes were controlled as confounding variables during the analysis of the relationship between PA and fatigue during pregnancy.

### Ethical approval

The research was performed in accordance with the Declaration of Helsinki and the protocol was approved by the Ethics Committee of Affiliated Maternity and Child Health Care Hospital of Nantong University (Y2021036). This project was registered in the Chinese Clinical Trial Registry (ChiCTR2100053966). Written informed consent was obtained from all participants. Participants were free to withdraw at any time.

## Results

**Figure 1:**
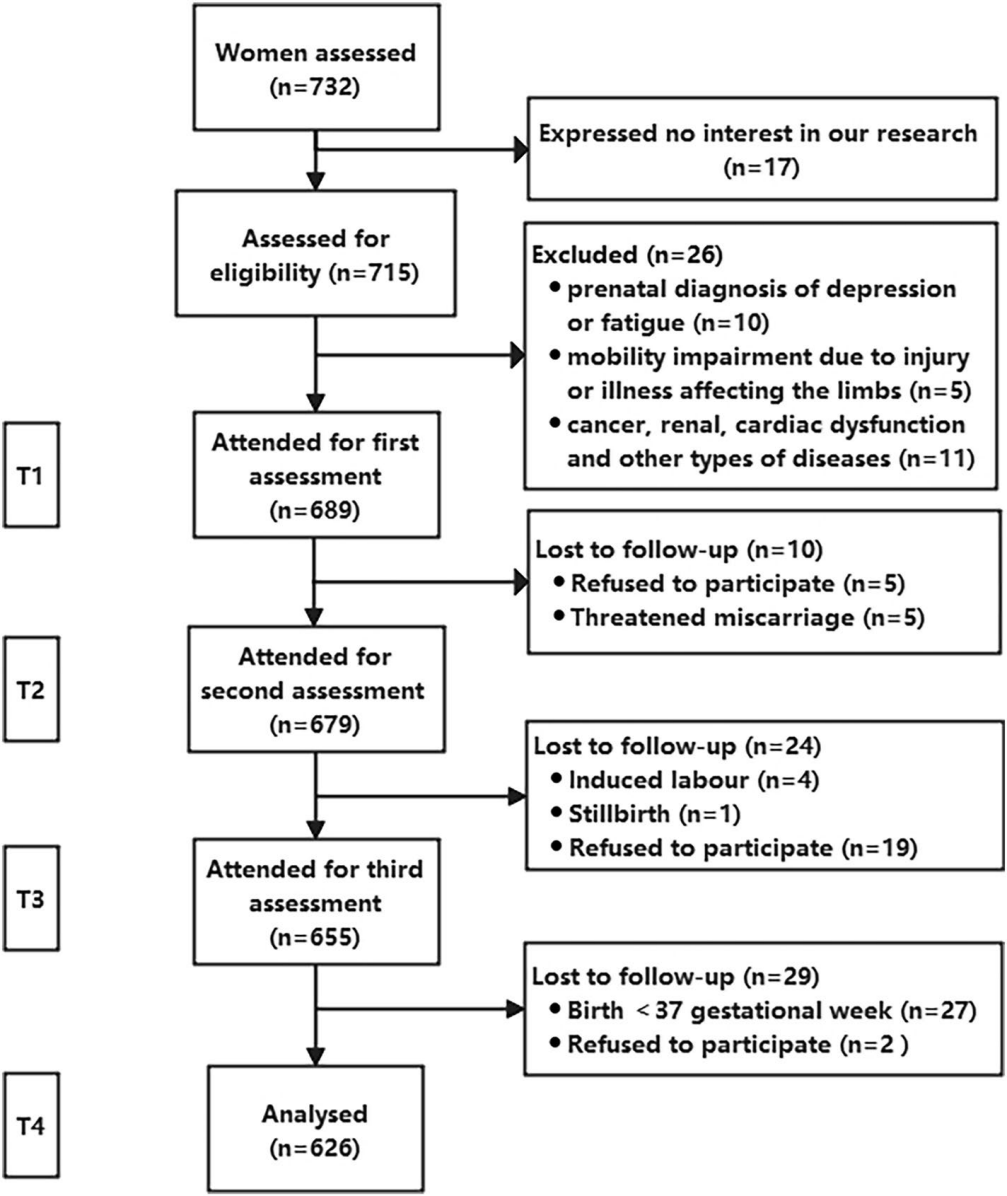
Flowchart of participants from eligibility to data analysis.

In total, 715 women were assessed for eligibility. Of this, 26 women did not meet the inclusion criteria, 63 dropped out during three follow-up surveys. Finally, a total of 626 participants were included in this longitudinal analysis (Fig. 1).

### Total physical activity

**Figure 2:**
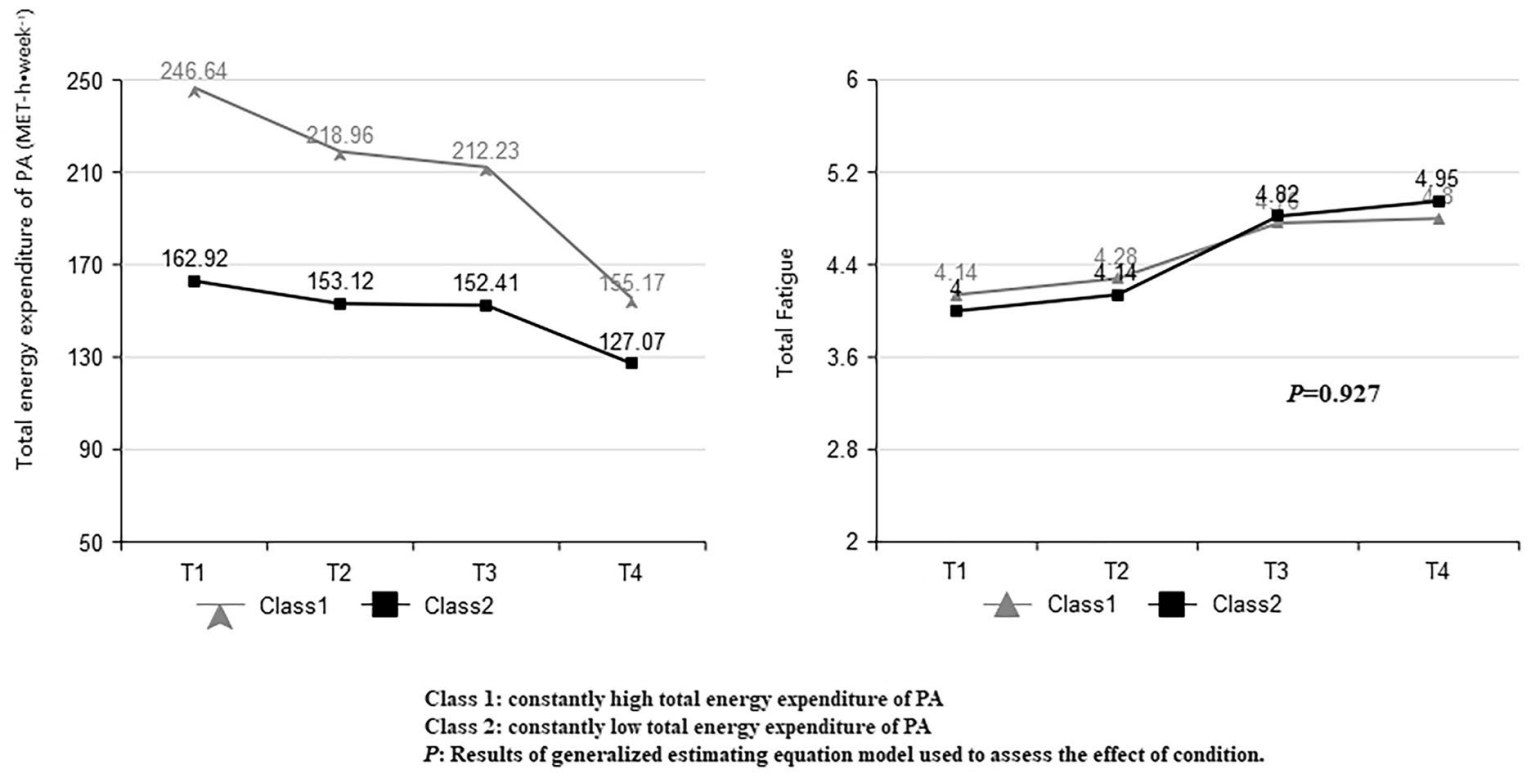
Total fatigue across trajectories of total energy expenditure of PA. Fatigue was not different between two groups based on total energy expenditure of PA (constantly high vs. constantly low).

The mean total energy expenditure during pregnancy was 184.35 ± 38.78 MET-h week^−1^. Table 1 showed the GMM of one to five fatigue trajectory classes. Based on the BIC values, the 4-class model fitted the PA trajectory data better than the 2-class, 3-class, and 5-class models. However, based on the entropy value, the 2-class model fitted the data better than the 3-class and 4-class models. The 2-class model was presented in Fig. 2. The majority of the sample of pregnant women (n = 374; 59.7%), based on their high overall total energy expenditure of PA, fell within a class that was labeled “constantly high”. The second class (n = 252; 40.3%) was labeled the “constantly low” class.

**Table 1:** Fit indices for the different GMM sequential models explored for total energy expenditure of PA across pregnancy.

C	AIC	BIC	aBIC	Entropy	*P* of LMR	*P* of BLRT	Class probability
1	25,580.589	25,620.543	25,591.969	-	-	-	-
2	24,627.568	24,694.159	24,646.536	0.925	0.000	0.000	59.7/40.3
3	24,374.635	24,467.862	24,401.190	0.809	0.000	0.000	38.3/22.5/39.1
4	24,311.539	24,418.084	24,341.887	0.819	0.029	0.000	37.9/15.8/31.5/14.9
5	24,353.299	24,459.843	24,383.646	0.916	0.000	0.000	38.0/1.3/12.1/45.7/2.9

*GMM,* growth mixture modeling; *PA,* physical activity; *C,* number of classes; *AIC,* akaike information criterion; *BIC*, Bayesian information criterion; *aBIC,* sample size adjusted BIC; *P of LMR,* p value of Lo-Mendel l-Rubin test; *P of BLRT,* *p* value of Bootstrap Likelihood Ratio Test; *Class probability*, proportion of sample classification.

Baseline characteristics of participants according to total energy expenditure of PA are presented in Table 2. GEE analyzed the relationship between PA trajectories and maternal fatigue. Total fatigue (*P* = 0.927), mental fatigue (*P* = 0.407) and physical fatigue (*P* = 0.537) showed no statistical difference among the two classes.

**Table 2:** Baseline patient characteristics between two groups based on total energy expenditure of PA.

	Class 1 (n = 374) $\overline{x} \pm s$/n (%)	Class 2 (n = 252) $\overline{x} \pm s$/n (%)	F/χ^2^	*P*
Women’s Age, year	28.22 ± 3.37	28.24 ± 3.78	0.004	0.949
Partners’ Age, year	29.24 ± 3.77	29.07 ± 3.89	0.324	0.570
Pre-pregnant BMI (kg/m^2^)	22.60 ± 3.55	22.19 ± 3.63	2.005	0.157
Partners’ BMI (kg/m^2^)	24.20 ± 3.15	24.58 ± 3.14	2.107	0.147
Location				
Urban	179 (47.9)	121 (48.0)	0.001	0.970
Countryside	195 (52.1)	131 (52.0)		
Women’s Education (years)				
≤ 13	42 (11.2)	83 (32.9)	45.968	0.000
13–17	306 (81.8)	161 (63.9)		
> 17	26 (7.0)	8 (3.2)		
Partners’ Education (years)				
≤ 13	61 (16.3)	88 (34.9)	35.111	0.000
13–17	286 (76.5)	160 (63.5)		
> 17	27 (7.2)	4 (1.6)		
Household income (RMB/month)				
< ¥5,000	156 (41.7)	123 (48.8)	3.071	0.080
≥ ¥5,000	218 (58.3)	129 (51.2)		
Occupation				
Jobless	4 (1.1)	117 (46.4)	18.654	0.000
In-paid job	370 (98.9)	135 (53.6)		
Sedentary employment hours (h/week)				
0	54 (14.4)	219 (86.9)	349.465	0.000
≤ 3	78 (20.9)	33 (13.1)		
˃ 3	242 (64.7)	0 (0)		
Moderate to vigorous employment hours (h/week)				
0	317 (84.8)	250 (99.2)	36.81	0.000
≤ 3	57 (15.2)	2 (0.8)		
˃ 3	0			
Parity				
Primipara	309 (82.6)	196 (77.8)	2.264	0.132
Multipara	65 (17.4)	56 (22.2)		
Gestational diabetes				
Yes	40 (10.7)	26 (10.3)	0.023	0.880
No	334 (89.3)	226 (89.7)		
IVF				
Yes	20 (5.3)	22 (8.7)	2.752	0.097
No	354 (94.7)	230 (91.3)		
Women’s sleep quality	4.75 ± 2.22	4.97 ± 2.46	1.453	0.228
Women’s depression	3.72 ± 3.36	3.82 ± 3.68	0.117	0.732

Class 1: constantly high total energy expenditure of PA; Class 2: constantly low total energy expenditure of PA.

If women were unemployed, their moderate to vigorous employment hours is 0, Sedentary employment hours is 0.

### Household physical activity

The selection of household PA trajectories followed a similar process as described above. Based on the entropy values, the 3-class model fitted the household PA trajectories data better than the 2-class, 4-class, and 5-class models. The 3-class model also offered more detailed and informative subgroup classification compared to the 2-class model. As a result, the 3-class model was chosen as the preferred approach for characterizing household PA trajectories. (eTable 1 in Supplement 1).

Based on GMM analysis, three household PA trajectory groups emerged as the most optimal for capturing the longitudinal data: constantly high household PA (n = 434; 69.3%), constantly low household PA (n = 38; 6.1%) and constantly medium household PA (n = 154; 24.6%).

**Figure 3:**
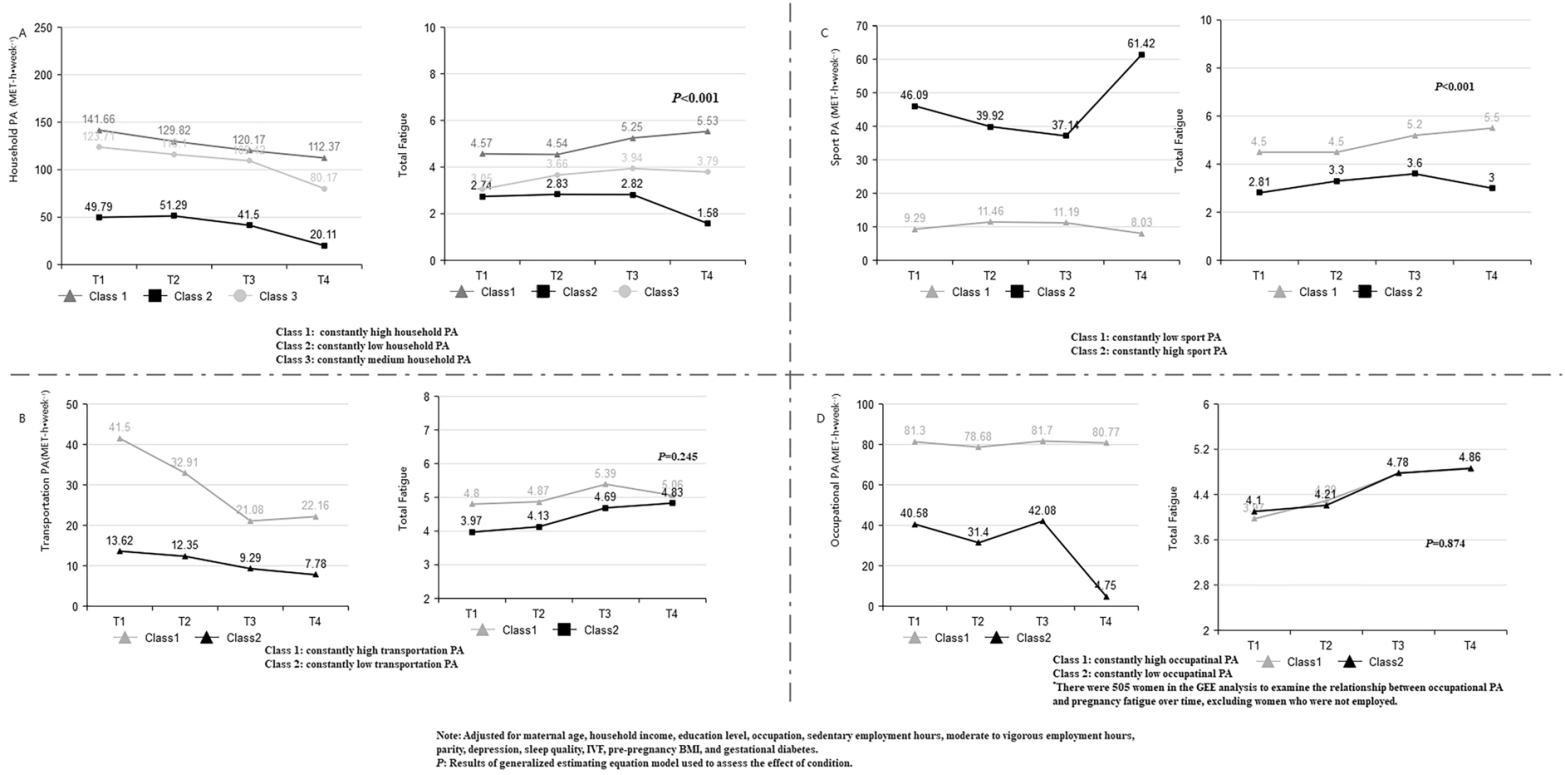
Total fatigue across trajectories of different types of PA. Note: Adjusted for maternal age, household income, education level, occupation, sedentary employment hours, moderate to vigorous employment hours, parity, depression, sleep quality, IVF, pre-pregnancy BMI, and gestational diabetes. *P*: Results of generalized estimating equation model used to assess the effect of condition. (**A**) Household PA was still an independent influencing factor for total fatigue after controlling for all covariates. (**B**) After adjustment for all covariates, the association between transportation PA and total fatigue was no longer observed. (**C**) Sport PA were still an independent influencing factor for total fatigue after controlling for all covariates. (**D**) No significant correlations were found between total fatigue and occupational PA.

Comparisons of baseline characteristics between the household PA groups were presented in eTable 2 in Supplement 1. Figure 3A demonstrated the three distinct trajectories of household PA and the results of the generalized estimating equations. Compared with women from the constantly low household PA group, women in the constantly high household PA group (*P* < 0.001) and the constantly medium household PA group (*P* < 0.001) had significantly higher total fatigue. Women in the constantly low household PA group had the lowest total fatigue (*P* < 0.001), mental fatigue (*P* < 0.001), and physical fatigue (*P* < 0.001), independently of maternal age, household income, education level, occupation, sedentary employment hours, moderate to vigorous employment hours, parity, depression, sleep quality, IVF, pre-pregnancy BMI, and gestational diabetes.

### Transportation physical activity

Based on the BIC values, the 5-class model fit the transportation PA trajectory data better than the 2-class, 3-class, and 4-class models. However, one class in both the 3-, 4- and 5-class model represented less than 5% of the total sample, indicating a spurious class. In comparison, the 2-class model demonstrated better fitted indices with better entropy values for transportation PA (eTable 3 in Supplement 1). Figure 3B illustrated transportation PA trajectory classes: constantly high transportation PA (n = 82; 13.1%) and constantly low transportation PA (n = 544; 86.9%).

Detailed baseline demographic characteristics and clinical data of the two groups were presented in eTable 4 in Supplement 1. Compared with the constantly low transportation PA group, pregnant women in constantly high transportation PA group showed higher total fatigue (*P* = 0.047) and mental fatigue (*P* = 0.021). After controlling for maternal age, household income, education level, occupation, sedentary employment hours, moderate to vigorous employment hours, parity, depression, sleep quality, IVF, pre-pregnancy BMI, and gestational diabetes, this association was no longer observed.

### Sport physical activity

Based on the BIC values, the 3-class model fitted the sport PA trajectory data better than the 2-class, 4-class, and 5-class models. However, the LMR-LRT test for the 3-class model was not significant. In comparison, the 2-class model demonstrated better fit indices due to its relatively stable population (eTable 5 in Supplement 1). Based on the GMM analysis, two distinct sport PA trajectory groups emerged as the most optimal model for characterizing the longitudinal data: constantly high sport PA (n = 471; 75.2%) and constantly low sport PA (n = 155; 24.8%). (Fig. 3C).

Detailed baseline characteristics were presented in eTable 6 in Supplement 1. As expected, compared with the constantly low sport PA group that maintained low sport PA levels, constantly high sport PA had lower levels of total fatigue (*P* < 0.001), mental fatigue (*P* < 0.001) and physical fatigue (*P* < 0.001). These associations persisted to be significant even after controlling for maternal age, household income, education level, occupation, sedentary employment hours, moderate to vigorous employment hours, parity, depression, sleep quality, IVF, pre-pregnancy BMI, and gestational diabetes.

### Occupational physical activity

Based on the BIC values, the 5-class model fitted the data better than the 2-class, 3-class, and 4-class models (eTable 7 in Supplement 1). However, the 3-, 4- and 5-class model contained a small proportion of participants (< 5%). Therefore, the 2-class model was selected as the best model (Fig. 3D).

The baseline demographic characteristics and clinical data of the two groups were shown in eTable 8 in Supplement 1. After excluding women who were not employed (n = 121), there were 505 women in the GEE analysis to examine the relationship between occupational PA and pregnancy fatigue over time. There were no significant correlations between the occupational PA and total fatigue (*P* = 0.874), mental fatigue (*P* = 0.689), and physical fatigue (*P* = 0.980) in the two groups.

## Discussion

In this longitudinal study of pregnant women, we characterized the trajectories of different types of physical activity during pregnancy. We identified two trajectories of total energy expenditure of PA: constantly high PA and decreasing PA. These profiles did not exhibit any significant association with maternal fatigue across the observed time period.

Compared with the constantly low household PA group, women in the constantly high household PA group and the constantly medium household PA group had significantly more severe fatigue over time. On the contrary, pregnant women in the constantly high sport PA group had lower levels of total fatigue than those in the constantly low sport PA group. These associations were still significant after controlling for confounding variables. But no significant association was observed for occupational PA and transportation PA.

### Total energy expenditure of PA

Enhancing the levels of physical activity among women of childbearing age during and after pregnancy, holds significant importance^27^. However, a considerable number of pregnant women fail to meet current physical activity recommendations, remaining inactive during pregnancy^28^. The mean total energy expenditure during pregnancy in our research was lower than a longitudinal Healthy Start study in Colorado^29^, but higher than a prospective cohort study in Vietnam^30^. This finding is consistent with prior research that physical activity typically declines throughout pregnancy^31^.

Previous research has shown that physical activity is a potentially promising method for preventing postpartum fatigue and depression, but very little scientific attention has been paid to exploring the association of PA and fatigue from a longitudinal perspective^32^. Using GMM, we identified two trajectories of total energy expenditure of PA over time. However, our studies found there was no association between total energy expenditure of PA and fatigue. Previous study recommended assessing physical activity in diverse situations including occupation, exercise, transportation and domestic life^33^. Therefore, we next explored the relationship between different types of PA and maternal fatigue.

### Household PA

In our population, household PA was the largest proportion of energy expenditure. We identified three trajectories of household PA over time. One household PA trajectory showed PA levels that remained constantly high across time. The second household PA trajectory that emerged showed constantly low across time. The third household PA trajectory reached a medium level. Even if energy expenditure of household PA decreases across gestation for all pregnancies, our results indicate that severe fatigue is influenced by a high energy expenditure on household PA.

Household PA includes preparing meals, taking care of children, watching TV or a video, sitting or reading and so on^34^. Earlier studies have indicated that women’s housework still takes more time than partners’ during pregnancy^35,36^. Housework division plays an important role in repeated muscle actions and fertility anxiety, which may lead to physical fatigue and mental fatigue individually^37^. High levels of sedentary time during pregnancy might induce the mental health problem of pregnant women^38^. Social support during pregnancy is necessary during pregnancy to reduce women’s housework-related fatigue. It is also important to advice that less sedentary lifestyle, such as reduce the time spent watching TV, can contribute to relieve mental fatigue.

In our research, the multiparas appeared more likely than the primiparas to have constantly low or medium household PA. A previous study found that their primiparas were higher unemployed than multiparas^39^. Primiparas were generally of a younger age and had poor level of knowledge and practice. They may spend more time at household PA, such as playing with pets, shopping (for food, clothes, or other items), watching TV and so on. This discovery is thought provoking and worth studying in the future.

### Sport PA

The maternal fatigue (total, physical and mental) differed between constantly high sport PA group and constantly low sport PA group in our study, suggesting that the low percentage of sport PA might be responsible for severe fatigue. The concepts of sport and physical activity are different. Sport PA includes walking slowly for fun, jogging, dancing, and so on, which is a subcategory of physical activity. It is widely accepted that women undertook high levels of sport activities to maintain physical and mental fitness^40^. There are also clear positive effects on depression, anxiety, and stress during pregnancy. Engaging in sports activities like running, swimming, playing ball, and walking may be beneficial ways to encourage people to be physically active and help relieve mental fatigue while ensuring sufficient rest^41^. Aerobic exercise has a beneficial impact on both physical fatigue and mental fatigue^42^. Women in constantly low sport PA group are likely strong candidates for early intervention. Special attention should be given to identifying and addressing barriers to physical activity during pregnancy, as well as addressing the preferences expressed by pregnant individuals for diverse and customizable activity options^43^.

Although current literature emphasizes the significance of exercise during pregnancy^44,45^, prevalence of sport PA during pregnancy is known to be generally low in China^15^. Exercise requirements increase in the latter trimester of pregnancy to more effectively suppresses body fat accumulation and weight gain^46^. In Poland, many pregnant women or greatly reduce their regular exercise routine because of the potential risks^26^. Our study found that there was a dramatic increase in sport PA in the fourth trimester, but energy expenditure was still below the recommended value specified in the guidelines^47^. This result may be related to the Chinese social and cultural background. The phenomena in this research hint that exercise intervention in the latter trimester of pregnancy can be better implemented.

### Transportation PA

This study could not possibly give a suggestion on transportation PA. There is limited evidence regarding the prospective association of transportation PA (e.g., walking or cycling to work) with mental health. But moderate transportation PA in fact provides important health benefits. Regular transportation PA helps improve heart and lung function, reduces the risk of heart disease, and lowers blood pressure^48^. With urbanisation, changes in transportation have reduced physical activity, such as walking or other modes^49,50^. It is documented that individuals in China tend to not view transportation PA as a viable means for enhancing their health. Previous study had found that there was a change towards less active transportation PA in early pregnancy compared to pre-pregnancy, because of increasing usage of private transportation^51^. Furthermore, these individuals perceive walking and cycling to work as obligatory rather than voluntary endeavors. Transportation PA could be beneficial for mental health, which is of great public health significance, and needs further investigation.

### Occupational PA

Finally, no relationship was found between occupational PA and maternal fatigue. The effect of occupational PA (e.g., heavy manual work, manual work, and standing occupation) on health is yielding conflicting results. However, most studies mainly concentrated on evaluating leisure-time physical activity as the primary exposure and claimed that occupational PA had a non-negligible health impact^52^. But other studies did not find that the occupational PA was associated with health benefits^53^. The scarcity and heterogeneity of existing literature on occupational PA may need for further studies that employ rigorous assessments of PA in the workplace. These studies would help elucidate the association in question.

Pregnancy period provides a challenge to the entire maternal body, every woman has a different experience of nausea or fatigue. It is also possible that maternal fatigue impact pregnancy physical activity. During the first trimester, maternal body undergoes many changes. Hormonal changes and metabolic during pregnancy can impact several organ systems, leading to symptoms that may arise as early as the initial weeks of pregnancy^54^. However, many of these discomforts tend to diminish as the pregnancy advances. Nausea and fatigue, in particular, are commonly reported to subside during the second and third trimesters. Then, many women find breathing difficult because of pressure on organs. And they may experience during this period include hemorrhoids, urinary incontinence, varicose veins and sleeping problems. Physical activity may be influenced by factors like those experiences of uncomfortable sensation.

## Limitations and future study

This study was one of the first to use the GMM approach to identify the trajectories of different types of PA across time. Unlike previous studies that employed categorical variables, our analysis utilized continuous physical activity variables. Moreover, we acknowledge that variations in the types of physical activity may exist across the populations under investigation.

The presented study is not free from limitations. Firstly, the sample of this study was only from one hospital, which failed to include women from other areas and did not explore geographical characteristics. Secondly, physical activity dropped worldwide during COVID-19, which should be taken into consideration by future researchers.

## Conclusion

The hypothesis of the present study posited that distinct PA trajectories have varying effects on maternal fatigue. In summary, using GMM, we found that higher household PA and lower sport PA were associated with higher fatigue during pregnancy. The displacement of household PA with sport PA in pregnant women necessitates focused attention physical activity guidelines and public health interventions. Such measures aim to mitigate the prevalence of fatigue and should provide direct and specific recommendations.

### Supplementary Information

Supplementary Information 1.

Supplementary Information 2.

Supplementary Information 3.

## Data Availability

The data and materials used for the current study are available from the corresponding author upon reasonable request.

## Abbreviations

AIC: Akaike information criterion
aBIC: Adjust Bayesian information criterions
BIC: Bayesian information criterions
BMI: Body mass index
CFI: Confirmatory fit index
DAG: Directed acyclic graph
FS-14: Fatigue scale-14
GEE: Generalized estimating equations
GMM: Growth mixture modeling
IVF: In vitro fertilization
MET: Metabolic equivalent of task
PPAQ: Pregnancy Physical Activity Questionnaire
WHO: World Health Organization
