# Supplementary material for: Physical activity trajectory during pregnancy and associations with maternal fatigue using a growth mixture modeling approach
Source: Sci Rep. 2024 Jan 10;14:1020. doi: 10.1038/s41598-024-51648-w (PMC10781982; doi:10.1038/s41598-024-51648-w)
Supplement: Supplementary file 1 — Supplementary Information 1. [file 41598_2024_51648_MOESM1_ESM.docx]

eTable 1. Fit indices for the different GMM sequential models explored for

household PA.

| C | AIC | BIC | aBIC | Entropy | *P* of LMR | *P* of  BLRT | Class probability |
| --- | --- | --- | --- | --- | --- | --- | --- |
| 1 | 22423.914 | 22463.868 | 22435.294 | - | - | - | - |
| 2 | 21333.058 | 21399.648 | 21352.025 | 0.866 | 0.000 | 0.000 | 75.4/24.6 |
| 3 | 21112.728 | 21205.954 | 21139.282 | 0.891 | 0.044 | 0.000 | 69.3/6.1/24.6 |
| 4 | 21045.010 | 21151.554 | 21075.357 | 0.877 | 0.003 | 0.000 | 25.7/60.3/4.6/9.3 |
| 5 | 20998.929 | 21118.792 | 21033.071 | 0.873 | 0.000 | 0.000 | 7.2/56.4/6.1/24.4/5.9 |

Note: GMM, Growth Mixture Modeling; PA, Physical Activity; C, number of classes; AIC, Akaike Information Criterion; BIC, Bayesian Information Criterion; aBIC, sample size adjusted BIC; *P* of LMR, p-value of Lo-Mendel l-Rubin test; *P* of BLRT, p-value of Bootstrap Likelihood Ratio Test; Class probability, proportion of sample classification.

eTable 2. Baseline patient characteristics between three groups based on household PA.

|  | Class 1 (n=434)  /n(%) | Class 2 (n=38)  ****/n(%) | Class 3 (n=154)  ****/n(%) | F/χ^2^ | *P* |
| --- | --- | --- | --- | --- | --- |
| Women’s Age, year | 28.15±3.44 | 28.80±3.81 | 28.32±3.75 | 0.665 | 0.524 |
| Partners’ Age, year | 29.05±3.53 | 31.17±7.16 | 29.02±3.29 | 5.639 | 0.004 |
| Pre-pregnant BMI (kg/m^2^) | 22.30±3.47 | 23.84±4.15 | 22.47±3.72 | 3.259 | 0.039 |
| Partners’ BMI (kg/m^2^) | 24.42±3.24 | 23.70±2.85 | 24.33±2.95 | 0.933 | 0.394 |
| Location  Urban  Countryside | 208 (47.9)  226 (52.1) | 22 (57.9)  16 (43.1) | 70 (45.5)  84 (54.5) | 1.890 | 0.389 |
| Women’s Education (years)  ≤13  13-17  >17 | 75 (17.3)  337 (77.6)  22 (5.1) | 9 (23.7)  27 (71.1)  2 (5.3) | 41 (26.6)  103 (66.9)  10 (6.5) | 7.509 | 0.111 |
| Partners’ Education (years)  ≤13  13-17  >17 | 94 (21.7)  319 (73.5)  21 (4.8) | 10 (26.3)  25 (65.8)  3 (7.9) | 45 (29.2)  102 (66.3)  7 (4.5) | 4.577 | 0.333 |
| Household income (RMB/month)  <¥5,000  ≥¥5,000 | 185 (43.6)  249 (57.4) | 15 (39.5)  23 (60.5) | 79 (51.3)  75 (48.7) | 3.885 | 0.143 |
| Occupation  Jobless  In-paid job | 84 (19.4)  350 (80.6) | 3 (7.9)  35 (92.1) | 34 (22.1)  120 (77.9) | 3.933 | 0.140 |
| Sedentary employment hours (h/week)  0  ≤3  ˃3 | 187 (43.1)  68 (15.7)  179 (41.2) | 10 (26.3)  10 (26.3)  18 (47.4) | 76 (49.4)  33 (21.4)  45 (29.2) | 12.650 | 0.013 |
| Moderate to vigorous employment hours (h/week)  0  ≤3  ˃3 | 399 (91.9)  35 (8.1)  0 | 29 (76.3)  9 (23.7)  0 | 139 (90.3)  15 (9.7)  0 | 10.010 | 0.007 |
| Parity  Primipara  Multipara | 358 (82.5)  76 (17.5) | 25 (65.8)  13 (34.2) | 122 (79.2)  32 (20.8) | 6.524 | 0.038 |
| Gestational diabetes  Yes  No | 41 (9.4)  393 (90.6) | 8 (21.1)  30 (78.9) | 137 (89.0)  17 (11.0) | 5.043 | 0.080 |
| IVF  Yes  No | 30 (6.9)  404 (93.1) | 1 (2.6)  37 (97.4) | 143 (92.9)  11 (7.1) | 1.084 | 0.581 |
| Women’s Sleep quality | 5.10±2.34 | 4.00±1.42 | 4.29±2.32 | 9.888 | 0.000 |
| Women’s Depression | 3.99±3.66 | 2.98±2.48 | 3.29±3.11 | 3.345 | 0.036 |

Note: Class 1: constantly high household PA; Class 2: constantly low household PA; Class 3: constantly medium household PA.

If women were unemployed, their moderate to vigorous employment hours is 0, Sedentary employment hours is 0.

eTable 3. Fit indices for the different GMM sequential models explored for transportation PA.

| C | AIC | BIC | aBIC | Entropy | *P* of LMR | *P* of  BLRT | Class probability |
| --- | --- | --- | --- | --- | --- | --- | --- |
| 1 | 19429.662 | 19469.616 | 19441.042 | - | - | - | - |
| 2 | 18751.743 | 18818.334 | 18770.711 | 0.803 | 0.000 | 0.000 | 13.1/86.9 |
| 3 | 18625.635 | 18718.861 | 18652.189 | 0.715 | 0.010 | 0.000 | 31.6/3.0/65.3 |
| 4 | 18587.266 | 18693.811 | 18617.614 | 0.738 | 0.070 | 0.000 | 52.9/6.9/0.2/40.0 |
| 5 | 18566.340 | 18686.202 | 18600.481 | 0.742 | 0.002 | 0.000 | 43.4/42.3/1.6/0.2/12.5 |

Note: GMM, Growth Mixture Modeling; PA, Physical Activity; C, number of classes; AIC, Akaike Information Criterion; BIC, Bayesian Information Criterion; aBIC, sample size adjusted BIC; *P* of LMR, p-value of Lo-Mendel l-Rubin test; *P* of BLRT, p-value of Bootstrap Likelihood Ratio Test; Class probability, proportion of sample classification

eTable 4. Baseline patient characteristics between two groups based on transportation PA.

|  | Class 1 (n=82)  /n(%) | Class 2 (n=544)  ****/n(%) | F/χ^2^ | *P* |
| --- | --- | --- | --- | --- |
| Women’s Age, year | 28.41±3.90 | 28.20±3.48 | 0.240 | 0.624 |
| Partners’ Age, year | 29.24±3.38 | 29.16±3.88 | 0.029 | 0.864 |
| Pre-pregnant BMI (kg/m^2^) | 22.62±3.35 | 22.41±3.62 | 0.238 | 0.626 |
| Partners’ BMI (kg/m^2^) | 24.58±3.09 | 24.32±3.16 | 0.491 | 0.484 |
| Location  Urban  Countryside | 37 (45.1)  45 (54.9) | 263 (48.3)  281 (51.7) | 0.297 | 0.636 |
| Women’s Education (years)  ≤13  13-17  >17 | 14 (17.0)  59 (72.0)  9 (11.0) | 111 (20.4)  408 (75.0)  25 (4.6) | 5.825 | 0.054 |
| Partners’ Education (years)  ≤13  13-17  >17 | 17 (20.7)  56 (68.3)  9 (11.0) | 132 (24.3)  390 (71.7)  22 (4.0) | 7.403 | 0.025 |
| Household income (RMB/month)  <¥5,000  ≥¥5,000 | 30 (36.6)  52 (63.4) | 249 (45.8)  295 (54.2) | 0.123 | 0.074 |
| Occupation  Jobless  In-paid job | 4 (4.9)  78 (95.1) | 117 (21.5)  427 (78.5) | 12.637 | 0.000 |
| Sedentary employment hours (h/week)  0  ≤3  ˃3 | 24 (29.3)  18 (22.0)  40 (48.8) | 249 (45.8)  93 (17.1)  202 (37.1) | 7.899 | 0.019 |
| Moderate to vigorous employment hours (h/week)  0  ≤3  ˃3 | 59 (72.0)  23 (28.0)  0 | 508 (93.4)  36 (6.6)  0 | 38.339 | 0.000 |
| Parity  Primipara  Multipara | 59 (72.0)  23 (28.0) | 446 (82.0)  98 (18.0) | 4.601 | 0.032 |
| Gestational diabetes  Yes  No | 8 (9.8)  74 (90.2) | 58 (10.7)  486 (89.3) | 0.062 | 0.493 |
| IVF  Yes  No | 5 (6.1)  77 (93.9) | 37 (6.8)  507 (93.2) | 0.056 | 0.520 |
| Women’s Sleep quality | 4.95±2.29 | 4.82±2.33 | 0.244 | 0.622 |
| Women’s Depression | 4.57±3.81 | 3.64±3.42 | 5.094 | 0.024 |

Note: Class 1: constantly high transportation PA; Class 2: constantly low transportation PA.

If women were unemployed, their moderate to vigorous employment hours is 0, Sedentary employment hours is 0.

eTable 5. Fit indices for the different GMM sequential models explored for sport PA.

| C | AIC | BIC | aBIC | Entropy | *P* of LMR | *P* of  BLRT | Class probability |
| --- | --- | --- | --- | --- | --- | --- | --- |
| 1 | 22683.244 | 22723.198 | 22694.624 | - | - | - | 1 |
| 2 | 21498.865 | 21565.455 | 21517.832 | 0.868 | 0.000 | 0.000 | 75.2/24.8 |
| 3 | 21301.761 | 21394.988 | 21328.316 | 0.904 | 0.061 | 0.000 | 22.0/5.8/72.2 |
| 4 | 21239.906 | 21346.450 | 21270.254 | 0.877 | 0.006 | 0.000 | 24.9/60.7/4.6/9.7 |
| 5 | 21202.877 | 21322.740 | 21237.018 | 0.762 | 0.455 | 0.000 | 38.0/35.0/4.6/8.1/14.2 |

Note: GMM, Growth Mixture Modeling; PA, Physical Activity; C, number of classes; AIC, Akaike Information Criterion; BIC, Bayesian Information Criterion; aBIC, sample size adjusted BIC; *P* of LMR, p-value of Lo-Mendel l-Rubin test; *P* of BLRT, p-value of Bootstrap Likelihood Ratio Test; Class probability, proportion of sample classification.

eTable 6. Baseline patient characteristics between two groups based on sport PA.

|  | Class 1  /n(%) | Class 2  ****/n(%) | F/χ^2^ | *P* |
| --- | --- | --- | --- | --- |
| Women’s Age, year | 28.22±3.52 | 28.26±3.61 | 0.014 | 0.905 |
| Partners’ Age, year | 29.09±3.57 | 29.43±4.49 | 0.931 | 0.335 |
| Pre-pregnant BMI (kg/m^2^) | 22.30±3.50 | 22.84±3.82 | 2.600 | 0.107 |
| Partners’ BMI (kg/m^2^) | 24.43±3.18 | 24.12±3.59 | 1.171 | 0.280 |
| Location  Urban  Countryside | 226 (48.0)  245 (52.0) | 74 (47.7)  81 (52.3) | 0.003 | 0.516 |
| Women’s Education (years)  ≤13  13-17  >17 | 84 (17.8)  362 (76.9)  25 (5.3) | 125 (20.0)  467 (74.6)  34 (5.4) | 5.689 | 0.058 |
| Partners’ Education (years)  ≤13  13-17  >17 | 102 (21.7)  345 (73.2)  24 (5.1) | 47 (30.3)  101 (65.2)  7 (4.5) | 4.830 | 0.089 |
| Household income (RMB/month)  <¥5,000  ≥¥5,000 | 203 (43.1)  268 (56.9) | 76 (49.0)  79 (51.0) | 1.661 | 0.197 |
| Occupation  Jobless  In-paid job | 93 (19.7)  378 (80.3) | 28 (18.1)  127 (81.9) | 0.211 | 0.646 |
| Sedentary employment hours (h/week)  0  ≤3  ˃3 | 207 (43.9)  77 (16.3)  187 (39.7) | 66 (42.6)  34 (21.9)  55 (35.5) | 2.640 | 0.267 |
| Moderate to vigorous employment hours (h/week)  0  ≤3  ˃3 | 433 (91.9)  38 (8.1)  0 | 134 (86.5)  21 (13.5)  0 | 4.103 | 0.043 |
| Parity  Primipara  Multipara | 386 (82.0)  85 (18.0) | 119 (76.8)  36 (23.3) | 2.006 | 0.157 |
| Gestational diabetes  Yes  No | 44 (9.3)  427 (90.7) | 22 (14.2)  133 (85.8) | 2.911 | 0.098 |
| IVF  Yes  No | 36 (7.6)  435 (92.4) | 6 (3.9)  149 (96.1) | 2.651 | 0.103 |
| Women’s Sleep quality | 5.07±2.31 | 4.13±2.22 | 19.817 | 0.000 |
| Women’s Depression | 3.94±3.60 | 3.21±3.07 | 5.223 | 0.023 |

Note: Class 1: constantly high sport PA; Class 2: constantly low sport PA

If women were unemployed, their moderate to vigorous employment hours is 0, Sedentary employment hours is 0.

eTable 7. Fit indices for the different GMM sequential models explored for occupational PA.

| C | AIC | BIC | aBIC | Entropy | *P* of LMR | *P* of  BLRT | Class probability |
| --- | --- | --- | --- | --- | --- | --- | --- |
| 1 | 25010.773 | 25050.727 | 25022.153 | - | - | - | - |
| 2 | 24521.167 | 24574.440 | 24536.341 | 0.994 | 0.050 | 0.000 | 12.3/87.7 |
| 3 | 24249.487 | 24316.078 | 24268.455 | 0.996 | 0.000 | 0.000 | 11.6/87.4/1.0 |
| 4 | 23922.019 | 24001.927 | 23944.780 | 0.989 | 0.188 | 0.000 | 11.0/1.1/75.9/12.0 |
| 5 | 23764.702 | 23857.928 | 23791.256 | 0.945 | 0.138 | 0.000 | 11.1/1.0/11.0/53.3/23.5 |

Note: GMM, Growth Mixture Modeling; PA, Physical Activity; C, number of classes; AIC, Akaike Information Criterion; BIC, Bayesian Information Criterion; aBIC, sample size adjusted BIC; *P* of LMR, p-value of Lo-Mendel l-Rubin test; *P* of BLRT, p-value of Bootstrap Likelihood Ratio Test; Class probability, proportion of sample classification.

eTable 8. Baseline patient characteristics between two groups based on occupational PA.

|  | Class 1 (n=77)  /n(%) | Class 2 (n=549)  ****/n(%) | F/χ^2^ | *P* |
| --- | --- | --- | --- | --- |
| Women’s Age, year | 29.51±2.61 | 28.05±3.61 | 0.014 | 0.905 |
| Partners’ Age, year | 30.20±2.66 | 29.03±3.93 | 0.931 | 0.335 |
| Pre-pregnant BMI (kg/m^2^) | 22.63±3.62 | 22.41±3.68 | 2.600 | 0.107 |
| Partners’ BMI (kg/m^2^) | 24.33±2.94 | 24.36±3.18 | 1.171 | 0.280 |
| Location  Urban  Countryside | 35 (45.5)  42 (54.5) | 265 (48.3)  284 (51.7) | 0.214 | 0.715 |
| Women’s Education (years)  ≤13  13-17  >17 | 6 (7.8)  63 (81.8)  8 (10.4) | 119 (21.7)  404 (73.6)  26 (4.7) | 11.106 | 0.004 |
| Partners’ Education (years)  ≤13  13-17  >17 | 15 (19.5)  54 (70.1)  8 (10.4) | 134 (24.4)  392 (71.4)  23 (4.3) | 5.946 | 0.051 |
| Household income (RMB/month)  <¥5,000  ≥¥5,000 | 34 (44.2)  43 (55.8) | 245 (44.6)  304 (55.4) | 0.006 | 0.519 |
| Occupation  Jobless  In-paid job | 0  77 (100) | 121 (22.0)  428 (78.0) | 21.037 | 0.000 |
| Sedentary employment hours (h/week)  0  ≤3  ˃3 | 6 (7.8)  11 (14.3)  60 (77.9) | 267 (48.6)  100 (18.2)  182 (33.2) | 61.431 | 0.000 |
| Moderate to vigorous employment hours (h/week)  0  ≤3  ˃3 | 65 (84.4)  12 (15.6) | 502 (91.4)  47 (8.6) | 3.902 | 0.048 |
| Parity  Primipara  Multipara | 58 (75.3)  19 (24.7) | 447 (71.4)  102 (18.6) | 1.609 | 0.218 |
| Gestational diabetes  Yes  No | 8 (10.4)  69 (89.6) | 58 (10.6)  491 (89.4) | 0.002 | 0.575 |
| IVF  Yes  No | 4 (5.2)  73 (94.8) | 38 (6.9)  511 (93.1) | 0.322 | 0.571 |
| Women’s Sleep quality | 4.78±2.37 | 4.84±2.32 | 19.817 | 0.000 |
| Women’s Depression | 3.71±3.18 | 3.77±3.53 | 5.223 | 0.023 |

Note: Class 1: constantly high occupational PA group; Class 2: constantly low occupational PA group. If women were unemployed, their moderate to vigorous employment hours is 0, Sedentary employment hours is 0.
