# Supplementary figures and images for: Physical activity trajectory during pregnancy and associations with maternal fatigue using a growth mixture modeling approach
Source: Sci Rep. 2024 Jan 10;14:1020. doi: 10.1038/s41598-024-51648-w (PMC10781982; doi:10.1038/s41598-024-51648-w)

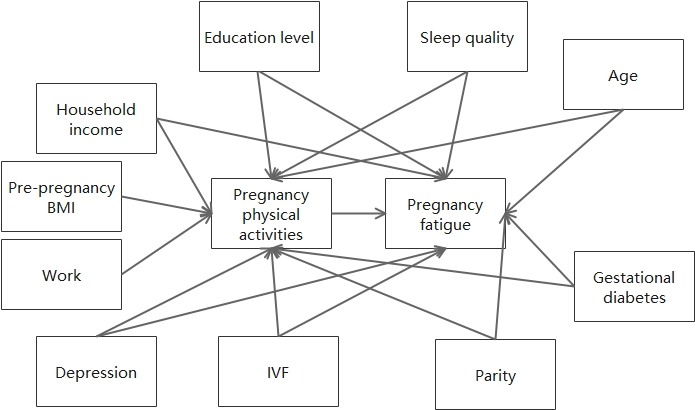

Supplement: Supplementary file 3 — Supplementary Information 3. [file 41598_2024_51648_MOESM3_ESM.jpg]
